# Economic evaluations of eye care services for Indigenous populations in high-income countries: a scoping review

**DOI:** 10.1186/s12939-024-02307-z

**Published:** 2024-11-09

**Authors:** Marcel Maziyar Nejatian, Andrei Sincari, Khyber Alam, Ian Li, Hessom Razavi

**Affiliations:** 1 Centre for Ophthalmology and Visual Science, The University of Western Australia, Perth, Australia; 2 Lions Eye Institute, 2 Verdun Street, Nedlands, Perth, WA 6009 Australia; 3 Metro North Health, Royal Brisbane and Women’s Hospital, Brisbane, Australia; 4 Department of Optometry, University of Western Australia, Perth, Australia; 5 School of Management and Marketing, Curtin University, Perth, Australia

**Keywords:** Indigenous eye health, Health inequities, Health economics, Cost-effectiveness

## Abstract

**Background:**

Indigenous people in high-income countries have worse eye health outcomes when compared to non-Indigenous people, contributing to ongoing socioeconomic disadvantage. Although services have been designed to address these disparities, it is unclear if they have undergone comprehensive economic evaluation. Our scoping review aimed to identify the number, type, quality, and main findings of such evaluations.

**Methods:**

MEDLINE, Embase, Web of Science, Cochrane Library Database, the National Health Service Economic Evaluation Database, EconLit, and relevant grey literature were systematically searched as per our pre-registered protocol. All economic evaluations of real or model services designed to meet the eye care needs of Indigenous populations in high-income countries were included. Two reviewers independently screened studies, extracted data, and assessed quality using the Quality of Health Economic Studies instrument.

**Results:**

We identified 20 studies evaluating services for Indigenous populations in Australia (*n* = 9), Canada (*n* = 7), and the United States of America (*n* = 4). Common services included diabetic retinopathy (DR) screening through fundus photographs acquired in local primary health care clinics (*n* = 7) or by mobile teams (*n* = 6), and general eye care through teleophthalmology (*n* = 2), outreach ophthalmology (*n* = 2) or an Indigenous health care clinic optometrist (*n* = 1). These services were economically favourable in 85% of comparisons with conventional alternatives, mainly through reduced costs of travel, in-person consults, and vision loss. Only four studies assessed the benefits of increased patient uptake. Only five included patient evaluations, but none integrated these into their quantitative analysis. Methodological issues included no stated economic perspective (*n* = 10), no sensitivity analysis (*n* = 12), no discounting (*n* = 9), inappropriate measurement of costs (*n* = 13) or outcomes (*n* = 5), and unjustified assumptions (*n* = 15).

**Conclusion:**

Several Indigenous eye care services are cost-effective, particularly remote DR screening. Other services are promising but require evaluation, with attention to avoid common methodological pitfalls. Well-designed evaluations can guide the allocation of scarce resources to services with demonstrated effectiveness and sustainability.

**Trial registration:**

Our scoping review protocol was pre-registered (Open Science Framework DOI: https://doi.org/10.17605/OSF.IO/YQKWN).

**Supplementary Information:**

The online version contains supplementary material available at 10.1186/s12939-024-02307-z.

## Background

Despite making up over 6% of the global population, Indigenous people experience political, economic, social, and health injustices contributing to their marginalisation within societies [1, 2]. While the majority live in low- to middle-income regions, Indigenous people in high-income countries also experience gross disparities in most indicators of well-being, including lower employment, income, education, and health status [1–3]. For example, the life-expectancy gaps between Indigenous and non-Indigenous populations are among the highest in the world in Canada (-12.5 years) and Australia (-10.0 years) [3].

Poor eye health is a well-described disparity experienced by many Indigenous populations in high-income countries. In Australia, extensive research, including two national surveys, have found higher rates of eye problems among Indigenous people when compared to the general population, including a three- to four-fold higher prevalence of vision loss in adulthood [4]. Studies in New Zealand [5], Canada [6, 7], and the United States of America (USA) [8], have similarly demonstrated eye health disparities among Indigenous communities. Many of these are attributed to reduced access to services, caused by geographical, financial, cultural, and other barriers [9, 10]. Accordingly, the Lancet Global Health Commission has identified the development of services that effectively prioritise and reach marginalised groups, such as Indigenous people, as a key priority in global eye health [11]. Such services, designed to improve access to eye care for Indigenous populations, will hereafter be referred to as Indigenous eye care services.

Within each country, limited resources are available to address a variety of competing health issues. To justify the use of scarce resources for Indigenous eye care services, it is vital that these have demonstrable value to individuals and society. Dunt et al. argues that such value can be demonstrated using three approaches: (1) health needs assessment based on disease epidemiology (e.g., prevalence); (2) economic evaluation; and (3) assessing the ability of a service to meet health performance benchmarks [12]. Notably, economic evaluations are increasingly recognised as essential tools for designing and implementing effective services which produce objective health benefits in a sustainable manner [13].

While Indigenous eye care services have value from a health needs and performance benchmark perspective, it is unclear whether they have undergone comprehensive economic evaluation [12]. We conducted the first known scoping review of this topic, aiming to identify the number and types of evaluations performed to date, and to summarise the reported economic impacts of specific services. This can guide policymakers and clinicians in making evidence-based decisions to support cost-effective services. We also appraised the methodological quality of these evaluations and identified knowledge gaps to inform future research.

## Methods

This scoping review followed the Preferred Reporting Items for Systematic Reviews and Meta-Analyses extension for scoping reviews (PRIMSA-ScR) [14] and the Joanna Briggs Institute (JBI) Guidance for Conducting Systematic Scoping Reviews [15]. No deviations were made from our pre-registered protocol (Open Science Framework DOI: https://doi.org/10.17605/OSF.IO/YQKWN). Two reviewers (M.M.N. and A.S.) independently screened reports for eligibility (title and abstract screening followed by full-text reviews), extracted data, and performed quality assessments. Discrepancies were resolved by consensus or with involvement of a third reviewer (H.R.).

### Eligibility criteria

Eligibility was defined using the Population, Intervention, Comparator, and Outcome (PICO) framework [14]:

- Population: wholly or partially Indigenous populations, as defined by the United Nations Permanent Forum on Indigenous Issues [1], within high-income countries, defined by the World Bank (Appendix A) [16].
- Intervention: real or model diagnostic, preventative, or therapeutic eye care service. Studies were excluded if there was no indication of how the service was designed and/or implemented to meet the needs of an Indigenous population.
- Comparator: any or no alternative service.
- Outcome: an economic evaluation, defined as any measure of service costs and/or service outcomes (i.e., health, monetary, or other benefits produced by the service) reported by a cost-minimisation analysis (CMA), cost-effectiveness analysis (CEA), cost-utility analysis (CUA), and/or cost–benefit analysis (CBA) [17]. Evaluations from any economic perspective were included. This refers to the viewpoint from which costs and outcomes are analysed (e.g., from the perspective of individual patients, the healthcare system, or society as a whole).

There were no restrictions on publication status or year of publication. The following were excluded: reviews, case studies, commentaries, conference abstracts, reports with no full-text access, and reports unavailable in English.

### Search strategy

A three-step strategy was conducted in consultation with a library and information scientist [15]. An initial search of MEDLINE using a preliminary strategy identified relevant reports. Index terms and keywords in the titles and abstracts were used to refine the strategy. MEDLINE (Ovid), Embase, Web of Science, Cochrane Library Database, the National Health Service Economic Evaluation Database (Ovid), and EconLit (EBSCO) were searched from inception to May 2023 using the refined strategy adapted for each database (Appendix B). No search limits or filters were applied. Database search records were imported into EndNote 20 and the deduplication function was used.

Grey literature was assessed through searches of Australian Indigenous HealthInfoNet, Vision 2020 Australia, Informit, the National Bureau of Economic Research, Canada’s Drug and Health Technology Agency, the Institute of Health Economics, the International Health Technology Assessment database, the International Agency for the Prevention of Blindness, and Google Scholar. Reference lists of all reviews and included reports were screened for additional reports.

### Data collection and quality assessment

The following were collected into standardised, pre-piloted data forms: study setting (country, rural versus urban), population, design (trial, observational, model-based) and methodology (type and economic perspective of analysis, time horizon, methods of costing and evaluating), service provided, comparator/s, and findings (costs, cost-effectiveness ratios, incremental cost-effectiveness ratios, benefit–cost ratios, and sensitivity analysis). Time horizon refers to the duration over which service costs and outcomes were analysed. Patient evaluations were recorded to capture the value of services from an Indigenous perspective, which is often underestimated or ignored in traditional economic analyses [18]. Methodological quality was assessed using the Quality of Health Economic Studies (QHES) instrument, a widely used checklist with demonstrated construct validity [19]. Two reviewers (M.M.N and A.S.) assessed each study against the 16 weighted criteria in this instrument [19], scoring them as ‘Yes’, ‘No’, or ‘Not Applicable’. Discrepancies were resolved by consensus or with involvement of a professor in economics (I.L.). The scores derived from this checklist were used to categorise the quality of studies as very poor (0–24), poor (25–49), moderate (50–74), and high (75–100).

### Data synthesis and analysis

Key study characteristics, findings, and quality were summarised through tabulation and narrative description. For comparability, all currencies were converted to international dollars using the purchasing power parity exchange rate for the year of pricing (or year of publication if pricing year was unreported) [20]. Prices were then inflated to 2023 using GDP implicit price deflators for the USA [21, 22]. A synthesis of issues with study quality and knowledge gaps was provided to inform future research practices and directions.

## Results

### Study selection and characteristics

**Fig. 1:**
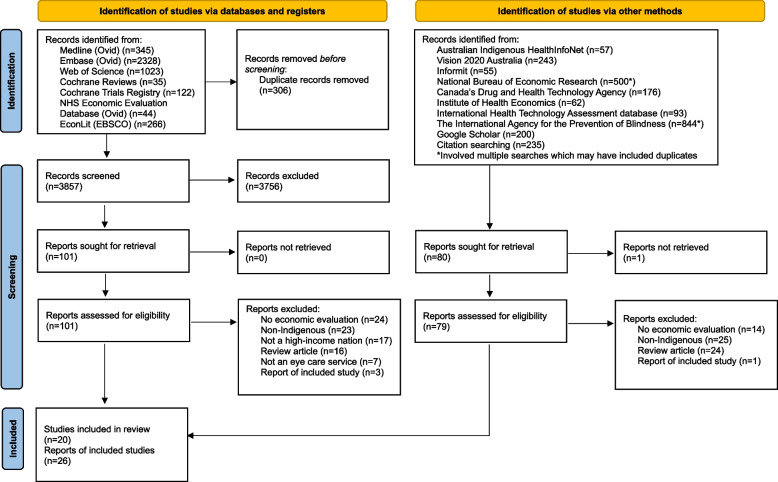
Preferred Reporting Items for Systematic Reviews and Meta-Analyses flow diagram of study selection

Of the 3857 unique database records, 101 underwent full-text review and 11 studies were included (Fig. 1). An additional 79 records from grey literature and citation searching underwent full-text review, of which nine studies were included. Reports excluded after full-text review were mostly evaluating non-Indigenous services (*n* = 48), review articles (*n* = 40), or lacked an evaluation of a service’s costs and/or outcomes as per our eligibility criteria (*n* = 38).

Key characteristics and findings of the 20 included studies are described in Table 1. Studies were published between 1990–1999 (*n* = 3), 2000–2009 (*n* = 6), and 2010–2019 (*n* = 11). They evaluated services for Aboriginal and Torres Strait Islander people in Australia (*n* = 9), First Nations people in Canada (*n* = 7), and Native Americans in the USA (*n* = 4). Some services also included non-Indigenous Australians (*n* = 6) and Canadians (*n* = 2). Studies were set in rural communities (*n* = 14), both urban and rural communities (*n* = 1), or nationwide (*n* = 3). All studies evaluating real services (*n* = 10) were observational in design. Model services were based on published epidemiological, cost, and/or treatment outcome data (n = 6), or represented simulated expansions of real services in geographical scale (*n* = 3) or duration (*n* = 1).

**Table 1:** Characteristics and findings of economic analyses of Indigenous eye care services in high-income countries

Author (year)	Country	Population & Setting	Study Design	Horizon (years)	Intervention	Comparator	Main Findings	Other Findings
**DR Screening (Primary Health Care Clinics)**								
Griffith (1993) [23]	USA	Native Americans in Yakima Indian Reservation (Rural^a^)	**CMA** – real service for 188 patients	2.75	Annual screening in Indigenous health clinic by (1) dilated direct ophthalmoscopy by GP OR dilated fundus photography read by offsite (2) general ophthalmologist or (3) retinal specialist. Ophthalmology consult for unreadable photos or worse than mild DR	Local ophthalmology consult	**Cost savings** per patient screened: (1) $105; (2) $71; (3) $57	Direct ophthalmoscopy was as sensitive as fundus photography
Maberley (2003) [24]	Canada	First Nation communities in Ontario (Rural)	**CEA and CUA** – Combined Monte Carlo and decision tree model of service for 650 patients with diabetes	5, 10 (cost, effect)	Annual screening by dilated fundus photography in unspecified clinic. Proliferative DR and macular oedema (prevalence from unpublished survey) undergo PRP which reduces blindness (based on RCT) leading to gain in QALYs. Higher uptake than comparator (assumption of 80% vs 55%) but less sensitive (80% vs 95%, based on published observational data)	Outreach retinal specialist and PRP if indicated	**Cost effective and dominant: cost savings** per patient of $125 ($115 vs $240) and detected more DR leading to 19% gain in QALYs from additional blindness avoided	Remained cost-effective under comprehensive sensitivity analysis, including 10,000 Monte Carlo simulations
Whited (2005) [25]	USA	Native Americans accessing the Indian Health Service (Nationwide)	**CEA** – Combined Monte Carlo and decision tree model of real service expanded for 81,000 patients	1	Annual screening in Indigenous health clinics by non-dilated fundus photography read by offsite optometrist. Proliferative DR (prevalence from published data in general population) undergo PRP which reduces blindness (based on RCT) leading to federal cost savings (based on published cost analysis). Optometry or ophthalmology consult for unreadable photos. Higher uptake than comparator (70% vs 53%, based on unpublished audit), similar sensitivity (89%) and specificity (97%), and 10% unreadable rate (based on published observational data)	Local optometry or ophthalmology consult and PRP as indicated	**Cost savings** per patient of $10 (pre-PRP). **Cost effective and dominant:** detected more proliferative DR leading to 13% less blindness, while remaining cheaper (post-PRP)	Remained cost-neutral or saving in 73% of 10,000 Monte Carlo simulations. Becomes less dominant if sensitivity ≤ 62% or screening rate ≤ 57%
FNQLHSSC (2013) [26]	Canada	4 First Nation communities in Quebec (Rural)	**CMA** – real service for 89 patients	1	Annual screening in Indigenous health clinics by dilated fundus photography read by offsite retinal specialist. Repeat screen in 4–6 months (moderate DR) or ophthalmology consult (more advanced DR, other pathology, unreadable photo). All consults assumed to get treatment for proliferative DR (actual treatment data not used)	Out-of-town (1) optometry or (2) ophthalmology consult and treatment as indicated	**Cost saving** compared to (1) optometry (savings per patient screened of $382 [$738 vs $1,120]), and (2) ophthalmology consult ($210 [$738 vs $948])	
Ellery (2014) [27]	Australia	Aboriginal and Torres Strait Islander people (Nationwide)	**CEA and CUA** – Combined Monte Carlo and Markov model of nationwide service	40	Annual screening in primary care clinics by non-dilated fundus photography, read by trained health professionals. Gold standard optometry or ophthalmology consult for unreadable photos and suspected DR. Ophthalmology follow-up for diagnosed DR and PRP if sight-threatening (severe non-proliferative DR, proliferative DR, or macular oedema). PRP reduces vision loss and blindness (based on RCT), leading to reduced healthcare costs (based on published surveys) and gain in QALYs	(1) No screening, (2) dilated ophthalmoscopy by GP with optometry or ophthalmology consult if suspected DR (lower sensitivity and specificity than intervention, based on meta-analysis), or (3) local optometry or ophthalmology consult and PRP as indicated	**Cost effective** compared to (1) no screening and (2) GP screening (incremental cost of $9,858 and $22,231 per QALY gained, respectively). Cheaper than (3) optometry or ophthalmology consult but **less effective** (consults cost an incremental $7,298 per QALY gained)	Remained cost-effective compared to (1) no screening under comprehensive sensitivity analysis, including 10,000 Monte Carlo simulations. Sensitivity analysis not performed for other comparators
Kanagasingam (2015) [28]	Australia	Aboriginal and Torres Strait Islander (35%) and non-Indigenous people in Western Australia and Queensland (Rural)	**CMA** – real service for 1,093 patients	0.8	DR screening in primary care clinics using fundus photography read by offsite ophthalmologist	Either (1) out-of-town or (2) outreach ophthalmology consult	**Cost saving** compared to (1) out-of-town consult (~ $302 savings per patient screened). **Not cost saving** compared to (2) outreach ($123-$153 vs $53)	Automatic DR grading of photos had a high sensitivity (81%) and specificity (80%) but was not used in the economic evaluation component of the study
Ballreich (2016) [29]	Australia	Aboriginal and Torres Strait Islander people in all rural areas of Australia (Rural)	**CEA** – Decision tree model of service for 45,197 patients	1	Annual screening in 189 Indigenous health clinics by dilated fundus photography, read by software. Unreadable photos (assumption of 10%) require outreach optometry consult, which has higher sensitivity (80% vs 90%) and specificity (70% versus 95%) based on published meta-analysis. Outreach ophthalmology consult for any DR and PRP if sight-threatening (severe non-proliferative DR, proliferative DR, or macular oedema [prevalence from published data in target population])	Outreach optometry consult (assumes equal screening rate). Outreach ophthalmology consult and PRP as indicated	**Cost savings** of $197 per patient screened ($299 vs $496) but **less effective** (detects 81% of DR vs 90%). Screening by optometry costs an incremental $7,545 per DR case detected	
**DR Screening (Mobile)**								
Martin (1998) [30]	Canada	First Nation communities in British Columbia (Unstated)	**CMA** – model service for 450 patients	1	Annual mobile screening by fundus photography read by offsite ophthalmologist	Out-of-town ophthalmology consult	**Cost savings** per patient screened of $325 ($194 vs $519)	
Jin (2004) [31]	Canada	22 First Nation communities in British Columbia (Rural^b^)	**CMA** – real service for 339 patients	1	Annual mobile diabetes care service, including screening by digital fundus photography read by offsite retinal specialist. Diabetes nurse educator measures body mass index, blood pressure, examines feet, point-of-care haemoglobin A1c, blood lipid profile, urine albumin-creatinine ratio, and provides management supervised by offsite endocrinologist	Travel to closest GP, clinical lab, diabetes education centre, ophthalmologist, and endocrinologist	**Cost savings** per patient of $263 ($1,572 vs $1,835)	
Ho (2006) [32]	Australia	Aboriginal and Torres Strait (92%) and non-Indigenous people in 32 communities in Northern Territory (Rural)	**CMA** – model of real service for 700 patients expanded to run for 7 years	7	Annual mobile screening by (1) one or (2) two teams (total number screened assumed to be same for both). Dilated fundus photography, read by offsite ophthalmologist. Diabetes education provided	Outreach ophthalmology	Discounted **cost savings** of (1) $111,737 or (2) $65,552, with capital costs paid back in 2.5 years or 4.3 years, respectively. Extrapolated cost-savings per patient screened of $23 or $13	(1) One and (2) two teams remained cost-saving until capital cost increased by 240% and 40%, respectively. Screening uptake increased from 41 to 75% during trial
Kim (2015) [33]	Canada	43 First Nation communities on Vancouver Island (Rural)	**CMA** – real service for 524 patients	1	Mobile screening by fundus photography read by offsite ophthalmologist. Diabetes education via pamphlets and audio files delivered by portable electronic devices	GP review and referral for out-of-town ophthalmology consult and OCT	**Cost savings** per patient screened of $55 ($575 vs $630)	
Kanjee (2017) [34]	Canada	First Nation and non-Indigenous people in 49 communities in Northern Manitoba (Rural^c^)	**CMA** – real service for 4,676 patients	6	Mobile screening by dilated fundus photography read by offsite retinal specialist. Diabetes education provided	Out-of-town ophthalmology consult	**Cost savings** per patient screened of $1,029. Total savings of $1,319,569 per year	
Stanimirovic (2019) [35]	Canada	First Nations people and low-income groups in Ontario (Urban and Rural)	**CEA** – Decision tree model of real service expanded for 28,500 patients	5	Annual mobile screening by dilated fundus photography and OCT read by offsite retinal specialist. Higher screening rate (80% vs 55%), sensitivity (95% vs 75%) and specificity (85% vs 82%) than comparator (based on ‘expert opinion’)	Local optometry or ophthalmology consult	**Cost savings** per patient screened of $78 ($56 vs $134). **Cost effective and dominant** strategy – detects 46% more DR cases while costing $2,217,341 less ($274 vs $957 per DR case detected)	
**General Eye Care Services**								
Kumar (2006) [36]	Australia	Aboriginal and Torres Strait Islander (~ 15%) and non-Indigenous people in Carnarvon (Rural)	**CMA** – real service for 118 patients	1	Once weekly half-day nurse-led teleophthalmology clinic in local hospital with tonometry, slit lamp with photography, and non-dilated fundus photography. Records reviewed and plan provided by offsite ophthalmologist within 24 h. Most (94%) consults were screening for conditions. Common conditions included glaucoma (48%), DR (36%), cataract (5%), and trauma (3%)	GP review and referral for (1) out-of-town ophthalmology consult, (2) outreach ophthalmology consult, or (3) outreach ophthalmology then out-of-town consult for further testing	**Cost saving** compared to (1) out-of-town (savings per patient of $189 [$319 vs $508]) and (3) outreach then out-of-town consults ($439 [$319 vs $ 758]). **Cost-neutral** compared to (2) outreach (-$10 [$319 vs $309])	Savings increased with higher clinic frequency and efficiency. Became more expensive compared to (2) outreach if equipment depreciated over 5 instead of 7 years
Razavi (2016) [37]	Australia	Aboriginal and Torres Strait Islander and non-Indigenous people across rural Western Australia (Rural)	**CMA** – model of real service expanded to include all eligible patients	1	Real-time teleophthalmology consults in GP, optometry, and hospital clinics. Number of patients eligible estimated from audit of pre-existing comparator services. Eligibility determined by two ophthalmologists and one optometrist based on severity and complexity of each patient’s condition and distance to comparator service vs. closest clinic with established telehealth expertise and equipment	(1) Outreach or (2) out-of-town ophthalmology consult	**Cost saving** compared to (1) outreach (savings per patient of $191 [$176 vs. $367]) and (2) out-of-town consult ($1,137 [$176 vs. $1313]). Saves healthcare system $499,617 annually	Estimates cost to equip rural GP, optometry, and hospital clinics with basic, advanced, or state-of-the-art teleophthalmology equipment: optometry clinics the most cost-effective option ($0 for basic, $363,473 for advanced, and $2,346,053 for state-of-the-art)
Turner A (2011) [38]	Australia	Aboriginal and Torres Strait Islander and non-Indigenous people accessing outreach ophthalmic services (Rural)	**CMA** – real outreach services	1	Outreach ophthalmic clinic consultations funded using fee for service model	Outreach services funded using salary-based model	**Cost neutral** per patient attendance (surgical + clinic) ($500 vs $771, *p* = 0.12)	Associated with 2.5-fold more cataract surgeries (*p* = 0.03) and 2.5-fold more clinic consults per service week (*p* = 0.02). Trend towards 40% lower waiting time (*p* = 0.19)
Turner B (2011) [39]	Australia	Aboriginal and Torres Strait Islander and non-Indigenous people accessing outreach ophthalmic services (Rural)	**CMA** – real outreach services	1	Outreach services providing integrated optometry and ophthalmic care (‘service integration score’ ≥ 50%). Score based on level and quality of coordination and communication between optometry and ophthalmic care in each area (determined through semi-structured interviews with key stakeholders)	Outreach services with no or poor integration	**Cost neutral** per attendance (surgical + clinic) ($632 vs $551, (*p* = 0.35)	Trend towards improved surgical output (1.9-fold more cataract operations, *p* = 0.13) and clinic output (1.4-fold more clinic consults, *p* = 0.20) per service week, and 42% lower waiting time (*p* = 0.19)
Jaworski (1996) [40]	USA	Native Americans accessing optometry at an Indian Health Service (Unstated)	**CBA** – real service	1	Federally funded optometry clinic integrated within Indigenous health clinic. Benefits based on amount insurance parties are billed for each service provided	No optometry service	**Cost effective** – benefit to cost ratio of 2.44	
**Others**								
Miller (2003) [41]	USA	Native American preschoolers on the Tohono O'Odam reservation (Rural^d^)	**CMA** – model service	Not stated	Preschool astigmatism screening in community health clinics using (1) autokeratometry, (2) autorefraction, or (3) photoscreening. Threshold for referral for diagnostic exam set to achieve 90% sensitivity for each method and the comparator (based on published observational data in target population). Number and cost of false positives calculated	Screening by VA	Screening by (1) autokeratometry and (2) autorefraction **cost saving** when ≥ 400 and 985 children screened, respectively. (3) Photoscreening never cost saving (more expensive and less sensitive than VA)	Acknowledges that, unlike interventions, screening by VA can detect vision loss from non-refractive causes
PwC (2015) [42]	Australia	Aboriginal and Torres Strait Islander people (Nationwide)	**CBA** – model services (based on published national rates of eye conditions, service utilisation, and service costs)	10	Nationwide expansion of services to eliminate avoidable vision loss. Expansion so (a) annual cataract surgery rate for Indigenous people equals national average; (b) all Indigenous people ≥ 40 years old with refractive error receive spectacles every two years; (c) all Indigenous people ≥ 40 years with diabetes undergo annual DR screening and PRP if needed; and (d) trachoma elimination programs continue. Employment of Aboriginal Health Workers as coordinators assumed to eliminate patient dropout. Reduced vision loss leads to societal, fiscal, and wellbeing benefits (based on published data)	Current services based on current annual cataract surgery, refractive error coverage, DR screening, and PRP rates, and no funding to continue trachoma elimination programs. High dropout (based on unpublished ‘field observations’) which incurs costs without benefits	**Cost effective** – Incremental net societal and fiscal benefits of $289 million and $18 million, respectively, with benefit to cost ratios of 2.55 and 1.09, respectively. Between 1,700 to 7,300 years lived with disability averted	Limited sensitivity analysis, including no variation in estimates of cost, benefit, or dropout rates

*DR* Diabetic retinopathy, *USA* United States of America, *CMA* Cost-minimisation analysis, *GP* general practitioner / primary care physician, *CEA* Cost-effectiveness analysis, *CUA* Cost-utility analysis, *PRP* Panretinal photocoagulation, *VA* Visual acuity, *RCT* Randomised Controlled Trial, *QALY* Quality-adjusted life years, *FNQLHSSC* First Nations of Quebec and Labrador Health and Social Services Commission, *OCT* Optical coherence tomography, *CBA* Cost–benefit analysis, *PwC* PricewaterhouseCoopers

^a^Four of six census tracts part of the reservation are classified as non-metropolitan using Rural–Urban Commuting Area codes [43, 44].

^b^Largely serves rural/remote communities across northern British Columbia [45].

^c^Majority of Manitoba’s Northern Health Region is rural [46].

^d^Two out of three census tracts part of the reservation are classified as non-metropolitan using Rural–Urban Commuting Area codes [43, 44].

**Fig. 2:**
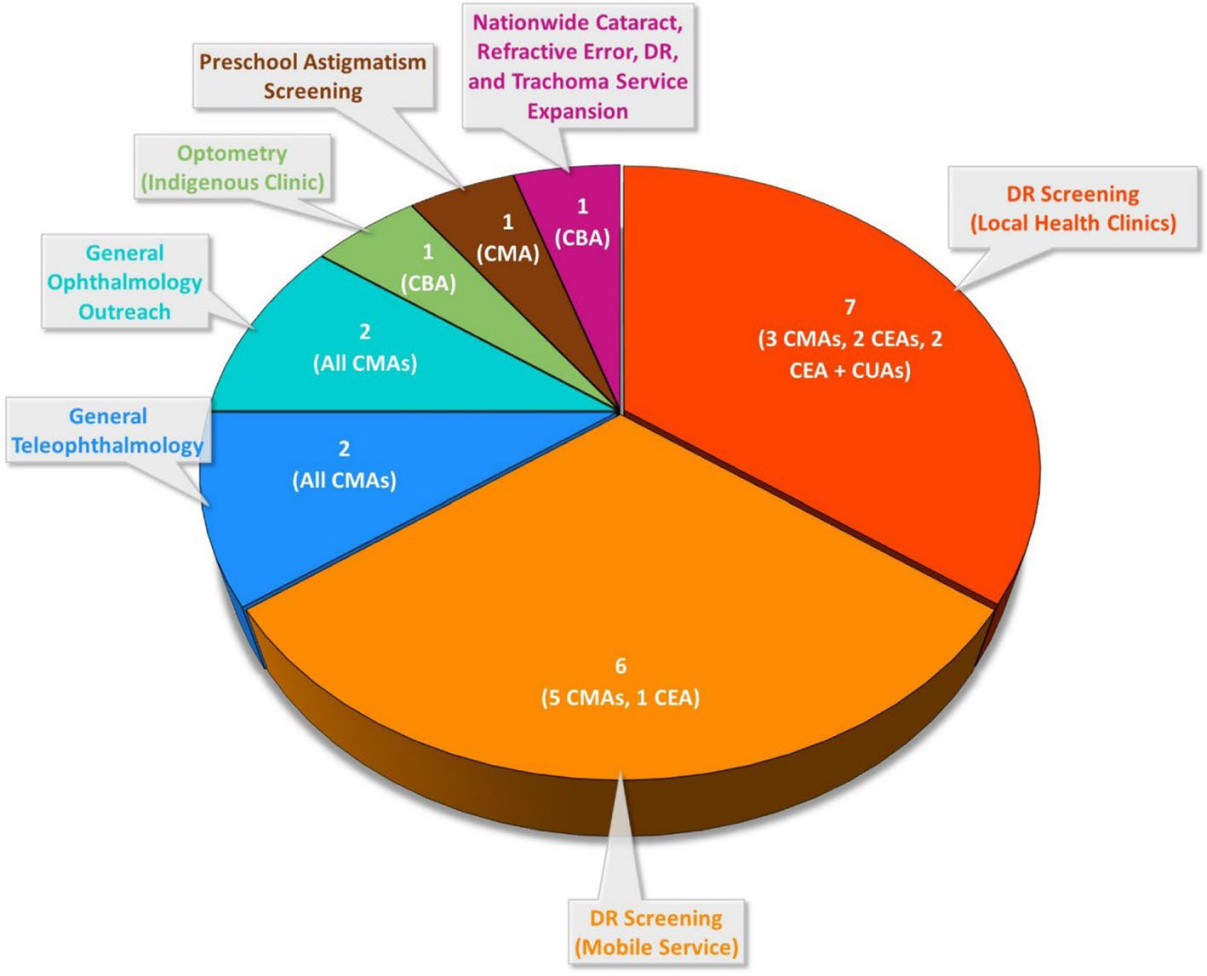
Number and type of economic evaluations of Indigenous eye care services. Cost-minimisation analyses (CMA), cost-effectiveness analyses (CEA), cost-utility analyses (CUA), and cost–benefit analyses (CBA) were all used

The most common Indigenous eye care services were diabetic retinopathy (DR) screening using fundus photographs acquired by trained staff physically located within local primary health care clinics [23–29] or by mobile teams [30–35] and graded by ophthalmologists who were located offsite at other facilities (Fig. 2). This was followed by general eye care services provided via teleophthalmology [36, 37], outreach ophthalmology [38, 39], or an Indigenous health clinic optometrist [40]. Only six of these services explicitly involved Indigenous community members and/or health care workers in their design [26, 31–33, 35, 42]. Across the 20 studies, there were 27 distinct comparisons between an Indigenous eye care service and a conventional alternative [23–37, 40, 42]. Some studies included multiple comparisons of different varieties of Indigenous eye care services and conventional alternatives. Three studies compared different varieties of Indigenous eye care services with each other rather than with a conventional alternative [38, 39, 41]. Evaluations adopted the economic perspective of the healthcare system [23, 24, 26–37, 41], federal government [25, 40], society [37, 42], or had an unclear perspective [38, 39].

### DR screening using fundus photography in local primary health care clinics

These studies were based in both rural and urban areas of Canada [24, 26], the USA [23, 25], and Australia [27–29]. Four primary comparators were used: screening by local ophthalmology or optometry services with no patient or staff travel costs [23, 25, 27], screening by out-of-town ophthalmology or optometry services associated with costs of patients travelling to visit them [26, 28], screening by outreach ophthalmology or optometry services associated with costs of staff travelling to the local town [24, 28, 29], or no screening [27].

Fundus photography in local primary health care clinics was cost-saving compared to screening by either local or out-of-town ophthalmology or optometry services, with savings per patient screened of $71 [23], $10 [25], ≥ $210 [26], and $302 [28]. Through improved patient uptake, Whited et al.’s model service within Indigenous primary health clinics in the USA also reduced DR-related blindness by 13% compared to screening by local ophthalmology or optometry services [25].

Fundus photography in local primary health care clinics was cost-saving compared to screening by rural outreach services in two CEAs [24, 29], but more expensive in one CMA [28]. Maberley et al.’s model service in Canada saved $125 per patient and, through higher uptake, led to a 19% gain in QALYs from avoided DR-related blindness [24]. While Ballreich et al.’s model service in Australia saved $197 per patient, it detected 10% fewer cases of DR due to the assumptions of equal uptake but lower sensitivity than outreach optometry [29]. Kanagasingam’s real service in rural Australia costed at least $70 more per patient, but there were substantial methodological issues with their study, including omission of nursing and clerical staff costs of the comparator [28].

Ellery et al.’s model service of DR screening using fundus photography in local primary health care clinics in Australia was cost-effective at reducing DR-related vision loss compared to no screening or screening by a general practitioner using direct ophthalmoscopy [27]. While cost-saving compared to screening by local optometrists and ophthalmologists, the service was less effective due to the assumed equivalent uptake but lower sensitivity.

### Mobile DR screening using fundus photography

There were four CMAs and one CEA in Canada [30, 31, 33–35] and one CMA in Australia [32]. The Canadian CMAs reported mobile services as cost-saving compared to patients travelling to out-of-town ophthalmologists, with savings per patient screened of $325 [30], $263 [31], $55 [33], and $1,029 [34] in the respective studies. The Canadian CEA reported that their model service saved $78 per patient while detecting 46% more DR than screening by local optometrists or ophthalmologists [35]. However, this study likely overestimated the cost-effectiveness of their service for two reasons: (1) mobile DR screening by fundus photography was assumed to be more sensitive and specific than screening by the comparator, which is inconsistent with published literature, and (2) the number of DR cases detected by the comparator was calculated assuming a screening rate of 55% (i.e., 15,675 patients screened), despite the cost being based on all 28,500 patients getting screened by an ophthalmologist. The Australian service saved $23 per patient compared to outreach ophthalmology, recouping the higher capital costs within 2.5 years [32].

### General eye care services

Two studies evaluated teleophthalmology in rural Western Australia [36, 37]. Kumar et al.’s nurse-led store-and-forward teleophthalmology service was cost-saving compared to out-of-town ophthalmology consults ($189 saved per patient) and cost-neutral compared to outreach ophthalmology [36]. Razavi et al. reported that real-time teleophthalmology would save $1,137 and $191 per patient compared to out-of-town and outreach ophthalmology services, respectively [37]. Through clinical audits, they determined that 15% and 24% of out-of-town and outreach consults, respectively, could be provided by teleophthalmology, which would save the healthcare system $499,617 annually. Each out-of-town consult avoided was also estimated to allow two extra days of work and, based on the nation’s average income, this would generate $443,023 in annual productivity savings for society.

Turner et al. conducted two evaluations of nine outreach ophthalmology services across Australia [38, 39]. Services funded using fee-for-service appeared cost-saving compared to those using a fixed-salary model ($500 vs $771 per clinic or surgical attendance), although this was not statistically significant (*p*= 0.12) [38]. These services were more efficient, with 2.5-fold higher clinic and surgical outputs (*p*= 0.02 and 0.03, respectively). Services well-integrated with outreach optometry had similar costs to those with poor integration but trended towards higher clinic and surgical outputs and lower waiting times [39].

Lastly, Jaworski found that integrating optometry within an Indigenous health clinic in the USA produced positive monetary returns, generating $2.44 in billings for every $1.00 in costs [40].

### Other services

Miller et al.’s CMA found that astigmatism screening of a preschool native American population by autokeratometry or autorefraction would begin resulting in cost savings after a minimum of 400 and 985 children, respectively, compared to screening by visual acuity [41]. These savings were attributed to a reduced number of false positive patients requiring follow-up eye exams, with each false positive exam costing $77 [41].

Lastly, a comprehensive CBA by PricewaterhouseCoopers (PWC) modelled the nationwide expansion of services to eliminate avoidable vision loss from cataract, refractive error, DR, and trachoma in Indigenous Australians [42]. Compared to preexisting services, the expansion produced a net incremental benefit of $298 million to society and $18 million to the government, with $2.55 and $1.09 in benefits, respectively, for every $1.00 spent expanding. Societal savings included the productivity gains from increased employment, at the national average income, of patients who would no longer have vision loss and the carers of such patients.

### Patient evaluations of services

Five of the 14 studies on real or modelled expansions of real services underwent patient evaluations (Table 2) [26, 28, 31, 35, 36]. These evaluations were all conducted through unvalidated questionnaires designed by study authors and had variable response rates. Over 90% of respondents were very satisfied or satisfied with the services [26, 28, 35, 36], would reuse them [26, 31, 36], and/or recommend them to others [31]. Reported benefits included convenience, particularly in relation to avoided travel and rapid access [26, 28, 31, 35, 36], increased awareness of eye health [26, 35], and the use of trusted local staff [26]. A minority using a general teleophthalmology service were concerned that it was less comprehensive and provided delayed advice compared to an in-person service [36].

**Table 2:** Patient evaluations of Indigenous eye care services

Author (year)	Country	Intervention	Response Rate (%)	Findings
Jin (2004) [31]	Canada	Mobile diabetes care service (including DR screening)	96	• 95% would reuse service • 95% would recommend service to others • 93% ranked service as more convenient than comparator
FNQLHSSC (2013) [26]	Canada	DR screening in local primary health care clinics	69	• 98% would reuse service • 98% very satisfied/satisfied with service • 92% found the use of local staff for the service acceptable • Reported benefits of service included proximity (85%), improved understanding of diabetes and DR (58%), use of entrusted local staff, service quality, and appointment flexibility
Kanagasingam (2015) [28]	Australia	DR screening in local primary health care clinics	17	• Mean satisfaction score of 9.7/10 • 20% of written feedback expressed appreciation of avoided travel
Stanimirovic (2019) [35]	Canada	DR screening in local primary health care clinics	-	• 92% rated service as excellent • 8% rated service as good • Reasons for not being screened prior to service: lack of awareness of DR (72%), cost (24%), or travel (4%)
Kumar (2006) [36]	Australia	General teleophthalmology service	41	• 98% would reuse service • 98% satisfied with service • 93% found service allowed quicker access to eye care • 88% had no privacy concerns with service • 74% not concerned about lack of direct contact with ophthalmologist • Complaints: small workspace, delayed ophthalmology advice, not as comprehensive as comparator

*DR* Diabetic retinopathy

### Quality of studies

**Fig. 3:**
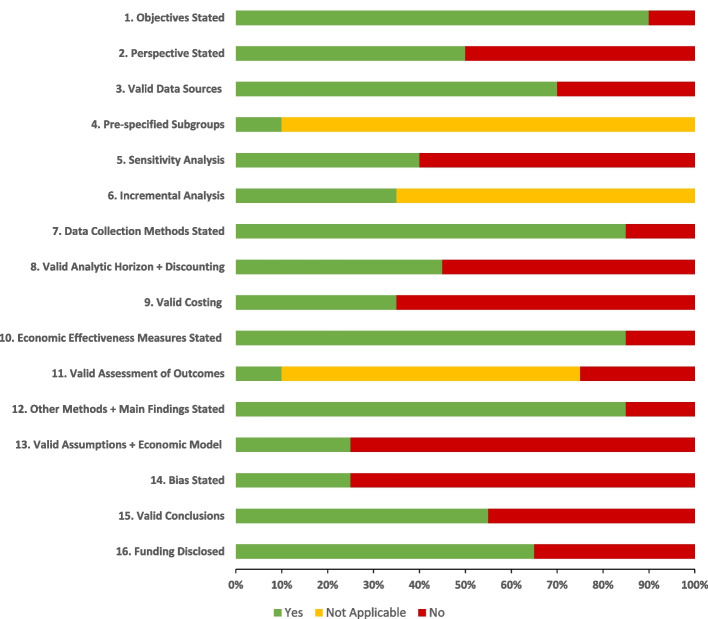
Proportion of studies scoring for each item on the Quality of Health Economic Studies (QHES) checklist

Figure 3 illustrates the proportion of studies meeting each item on the QHES checklist, with individual study scores detailed in Supplementary Table 1. Five studies were high quality [24, 25, 27, 37, 42], eleven were moderate [26, 29, 32–36, 38–41], three were poor [23, 30, 31], and one was very poor [28]. Most studies presented their objectives, methods, and findings clearly (QHES items 1, 10, and 12) and used appropriate sources for costs (real expenditures or local data) and health outcomes (randomised controlled trials) (items 3 and 7).

Ten studies failed to explicitly state their economic perspective (item 2) and 12 did not conduct a sensitivity analysis (item 5). Eight annuitized capital cost without discounting [28–31, 33, 34, 36, 41], one did not discount any costs beyond one year [23], and two used a limited timeframe potentially missing some health outcomes of their service [25, 42] (item 8). Supplementary Table 2 outlines the types of costs and outcomes considered in each study. Thirteen studies had inappropriate or unclear methods for calculating costs (item 9). Specifically, three omitted capital costs completely [23, 26] or partially [42], five were unclear about the components of capital costs and whether these were annuitized [28, 33, 34, 38, 39], three were unclear about components of their operating costs [31, 38, 39], four omitted costs of some or all staff [24, 26, 30, 41] or treatment [24], and one included costs for patients not using their service [35]. Five of the seven non-CMA studies used invalid methods for assessing outcomes (item 11), including overestimating blindness avoided from DR screening [24, 27], overestimating service income [40], and calculating the cost per case of DR detected without including the savings associated with reduced vision loss once these cases are treated [29, 35].

The main assumptions and/or choice of economic model were unjustified in 12 out of 13 CMAs (item 13). Specifically, two omitted all capital costs [23, 26], four used a comparator which was more comprehensive than their service [31, 33, 36, 41], and two could not determine if their findings were confounded by other variables [38, 39]. Six of the eight CMAs of DR screening by fundus photography omitted the costs of consults for unreadable and abnormal photos [28, 30–34]. Conversely, three of the seven non-CMA studies had major assumptions that were unjustified [27, 29, 35]. Ellery et al. [27] and Ballreich et al. [29] assumed that uptake of their model DR screening services would be equal to conventional screening, despite increased availability being the main purpose of their services. This led to their services detecting fewer cases of DR. Stanimirovic et al. made assumptions likely leading to overestimation of the cost-effectiveness of their service (Supplementary Table 2) [35].

## Discussion

Across all studies, we identified 27 comparisons between a service for Indigenous populations in Australia, Canada, or the USA and a conventional alternative. Indigenous eye care services were economically favourable in 23 (85%) of these comparisons, despite most omitting key benefits of culturally tailored care including increased patient uptake and value from an Indigenous perspective. Several common methodological pitfalls were identified, which should be avoided in future evaluations.

The primary economic value of services identified in our review arose from reduced costs of travel and in-person consults. Up to two thirds of Indigenous people in Australia, Canada, and the USA live outside major cities [47]. Traditionally, accessing eye care either requires patient travel to major cities or outreach services, both of which have high logistical costs borne by the health care system and patient [48–50]. Nine studies found that DR screening through fundus photographs, acquired by local primary health care clinics or mobile teams and graded offsite, led to health care savings by avoiding costs of patient travel [26, 28, 30, 31, 33, 34] or outreach services [24, 29, 32]. While the remaining four studies on DR did not consider travel costs, they found that these screening methods were cost-saving through avoiding expensive in-person optometry or ophthalmology consults [23, 25, 27, 35]. General teleophthalmology services in rural Australia also led to health care savings through reduced patient travel [36, 37] and outreach service expenses [37]. Many studies may have underestimated the savings from local Indigenous eye care services by not accounting for indirect costs associated with patient travel, such as the travel costs of companions (included in only two studies [26, 33]) and the productivity losses for patients when travelling (included in one study [37]). For example, the inclusion of productivity losses from patient travel led to an 89% increase in savings in the single study that analysed this [37].

The services identified adopted evidence-based strategies to improve accessibility and uptake by Indigenous populations, including local delivery of care, integration within Indigenous health clinics, and use of Indigenous health workers [9]. The positive patient evaluations of services further indicates that these strategies would improve uptake relative to conventional care. Despite this, only four studies evaluated the impact of increased patient uptake [24, 25, 35, 42], with the remainder potentially underestimating the cost-effectiveness of their services. For instance, among the eleven studies comparing local DR screening [24, 26, 28–34] or teleophthalmology [36, 37] to out-of-town or periodic outreach services, only Maberley et al. [24] included uptake as a variable, where higher screening rates increased QALYs by 19%. Among the four studies evaluating DR screening in local Indigenous health care clinics [23, 25, 26, 29], only Whited et al. [25] explored the benefit of integrated care on patient uptake. In their analysis, higher uptake contributed to reduced DR-related blindness and associated savings on health care and social welfare costs and increased income tax revenue. Lastly, among six services recruiting Indigenous health workers [29, 32, 33, 38, 39, 42], only PWC’s [42] evaluation explored the economic benefit of this strategy, whereby service coordination by Aboriginal Health Workers was modelled to increase patient uptake and reduce drop-out. This contributed to reduced vision loss with associated savings in health care and social welfare costs and increased societal income and tax revenue. To fully capture the economic benefits of Indigenous health programs, future studies must include patient uptake within their evaluations.

All studies adopted traditional methods of economic evaluation, which may underestimate the value of services from an Indigenous perspective [18]. While traditional evaluations focus on value derived from the health gain of individuals, Indigenous concepts of health extend beyond the individual to include the health and empowerment of their community, connections to land, and cultural security [18, 51]. For instance, an Indigenous-designed service is valued more by Indigenous people than one which delivers equal individual health benefits in a less culturally sensitive manner [52]. Despite this, none of the six studies involving Indigenous people in service design analysed the economic value of this collaboration [26, 31–33, 35, 42]. The New South Wales government recently published strategies to overcome these recognised limitations of traditional evaluations, such as the use of contingent valuation methods to quantify the value of different services from the perspective of Indigenous communities [53]. Other strategies which could be adopted by future studies include Indigenous-specific discrete choice experiments and health-related quality of life measures [51, 52].

Several other methodological issues limited the quality of studies included in our review. Future studies should include the economic perspective of their evaluation, sensitivity analyses, discounting items beyond one year, and clear methods for estimating capital and operating costs including sources used, components included, and annuitization. An economic evaluation checklist, which none of the studies explicitly used, may help ensure such essential items are included and avoid other methodological pitfalls [19]. As different eye care services are unlikely to have identical outcomes, CMAs should be avoided. Lastly, any study of screening or diagnostic services should use accurate estimates of sensitivities and specificities, as these significantly impact economic outcomes, as was the case in Stanimirovic et al.’s evaluation [35].

Our review also highlights the need for evaluations of multiple services yet to be analysed. Firstly, while DR screening through offsite grading of fundus photographs has been evaluated, automated grading through artificial intelligence can provide a cheaper, timelier service while maintaining acceptable accuracy [54, 55]. This could more easily be expanded nationwide to achieve universal screening, which should lead to extensive savings particularly for Indigenous people who have less access to screening despite a higher prevalence of DR [55]. Indeed, a study published in 2024 predicts that such screening methods among Indigenous Australians would generate a net societal benefit of $509 million dollars [56]. Evaluations of real-world applications of such services should be conducted to confirm these findings. A review by Burn et al. identified 37 studies on other, non-DR related service delivery models designed to improve access to eye care for Indigenous populations in high-income countries. [9] Most have not undergone economic evaluation, including mobile general ophthalmology services in Australia [57] and Taiwan [58], integration of optometry care within Indigenous health care clinics within Australia [59, 60], integration of preoperative and/or postoperative cataract assessments within Indigenous health care clinics [61] or optometry services [62], Indigenous spectacle subsidy schemes [59, 60], trachoma control programs [63], or culturally tailored health promotion activities [63–65]. While many of these have demonstrated potential to improve access, evaluations are needed to identify which represent the best value for money. Future studies should particularly focus on services which target the most common causes of vision loss in Indigenous populations, such as refractive error, cataract, and diabetic retinopathy [4–8]. Lastly, evaluations of Indigenous populations in other countries with documented disparities in eye health should be considered, such as those living in New Zealand, Taiwan, and Greenland [66].

### Limitations

The lack of a meta-analysis prevented a statistical evaluation of the cost-effectiveness of services. However, given the limited number of heterogenous studies, a meta-analysis is unlikely to provide meaningful results [67]. Nine of the 20 studies included were conducted prior to 2010 and may be less relevant to modern times given changes to the costs of providing services. We minimised the impact of this through providing inflation adjusted results. Excluding the four poor and very poor-quality studies may have improved the relevance of findings summarised in our review. However, as a scoping review, we aimed to provide a comprehensive overview of all evaluations performed and highlight common issues with study quality that should be addressed in future research. Lastly, during the systematic search, we identified five studies which analysed the cost of real Indigenous eye care services without including outcomes or a comparator (Appendix C). Including cost-only studies may have provided useful data about these additional services but was beyond the scope of our review. Furthermore, such partial economic evaluations have limited value in decision-making, as they provide no indication of the value for money of a service [17].

## Conclusions

Our review identified a variety of cost-saving and/or cost-effective DR screening, general ophthalmology, and optometry services for Indigenous populations in Australia, Canada, and the USA. Services that improve access to DR screening were particularly well explored and could substantially reduce avoidable vision loss among Indigenous populations. Future evaluations should include the economic impact of improved uptake and Indigenous concepts of health, while avoiding common methodological pitfalls, particularly those related to the assessment of costs and outcomes.

## Supplementary Information

Supplementary Material 1.

Supplementary Material 2.

## Data Availability

The data supporting the conclusions of this article are included within the article and its additional files.

## Abbreviations

DR: Diabetic retinopathy
USA: United States of America
JBI: Joanna Briggs Institute
CMA: Cost-minimisation analysis
CEA: Cost-effectiveness analysis
CUA: Cost-utility analysis
CBA: Cost-benefit analysis
QHES: Quality of Health Economic Studies
PWC: PricewaterhouseCoopers
