# Supplementary material for: Economic evaluations of eye care services for Indigenous populations in high-income countries: a scoping review
Source: Int J Equity Health. 2024 Nov 9;23:232. doi: 10.1186/s12939-024-02307-z (PMC11549826; doi:10.1186/s12939-024-02307-z)
Supplement: Supplementary file 1 — Supplementary Material 1. [file 12939_2024_2307_MOESM1_ESM.docx]

**Appendices**

**Table of Contents**

[**Appendix A:** List of high-income countries as defined by the World Bank (gross national income per capita of ≥US$13,205)^1^ 2](#_Toc167868409)

[**Appendix B:** Search Strategy 3](#_Toc167868410)

[**Appendix C:** Summary of cost-only studies on Indigenous eye care services identified during our systematic search. 13](#_Toc167868411)

[**References** 14](#_Toc167868412)

# **Appendix A:** List of high-income countries as defined by the World Bank (gross national income per capita of ≥US$13,205)^1^

| Region^a^ | Countries |
| --- | --- |
| Africa | Seychelles |
| Asia | Bahrain, Brunei Darussalam, Cyprus, China (Macao and Hong Kong Special Administrative Regions, and Taiwan), Israel, Japan, Republic of Korea, Kuwait, Oman, Qatar, Saudi Arabia, Singapore, United Arab Emirates |
| Latin America and the Caribbean | Aruba, Antigua and Barbuda, The Bahamas, Barbados, British Virgin Islands, Cayman Islands, Chile, Curaçao, Panama, Puerto Rico, Sint Maarten (Dutch part), Saint Kitts and Nevis, Saint Martin (French part), Trinidad and Tobago, Turks and Caicos Islands, United States Virgin Islands, Uruguay |
| Europe | Andorra, Austria, Belgium, Channel Islands, Croatia, Czech Republic, Denmark, Estonia, Faroe Islands, Finland, France, Germany, Gibraltar, Greece, Hungary, Iceland, Ireland, Isle of Man, Italy, Latvia, Liechtenstein, Lithuania, Luxembourg, Malta, Monaco, Netherlands, Norway, Poland, Portugal, Romania, San Marino, Slovakia, Slovenia, Spain, Sweden, Switzerland, United Kingdom |
| North America | Bermuda, Canada, Greenland, United States of America |
| Oceania | America Samoa, Australia, French Polynesia, Guam, Nauru, New Caledonia, New Zealand, Northern Mariana Islands |

^a^ Defined by the United Nations Standard Country or Area Codes for Statistical Use.^2^

# **Appendix B:** Search Strategy

| Source | Term | | Results |
| --- | --- | --- | --- |
| DATABASES: |  |  |  |
| Ovid MEDLINE ®  <1946 to 13/06/2023> | 1 | exp indigenous peoples/ or minority groups/ or exp oceanians/ or caribbean people/ or indians, south american/ or exp american indian/ or alaska native/ or "health disparate minority and vulnerable populations"/ | 53473 |
|  | 2 | (indigenous or native or aborigin* or first nation* or first people* or islander or trib* or elder or ancest* or village* or ethnic minority).tw. | 475074 |
|  | 3 | (ATSI or Torres Strait* or Australoid or closing the gap).tw. or health services, indigenous/ | 7363 |
|  | 4 | (Polynesian or Maori or Hawaiian).tw. | 9394 |
|  | 5 | (Indian* or Amerind or Pima or Akimel O'otham or Navajo or Navaho or Dine).tw. not India/ | 70389 |
|  | 6 | (Inuit* or Inuk or Kalaallit or Inupiat or Aleut or Eskimo or Metis).tw. | 3039 |
|  | 7 | or/1-6 | 567462 |
|  | 8 | exp ophthalmology/ or exp optometry/ or exp ophthalmologic diagnostic techniques/ or exp ophthalmologic surgical procedures/ or exp intraocular injection/ or exp eye diseases/ or exp eye injuries/ | 736350 |
|  | 9 | (ophthal* or optometr*).tw. | 125887 |
|  | 10 | (eye or eyes).tw. | 411238 |
|  | 11 | (vision or visual* or sight or blindness).tw. | 865748 |
|  | 12 | (teleophthalmolog* or tele ophthalmolog*).tw. | 461 |
|  | 13 | (amblyopia or aphakia or cataract or cornea* or dry eye or glaucoma or macular degeneration or macular oedema or macular edema or onchocerciasis or onchocerciases or optic or orbital or pterygium or retina or retinal or retinopathy or river blindness or trachoma or trichiasis).tw. | 592758 |
|  | 14 | (astigmatism or hyperopi* or hypermetropi* or myopi* or presbyopia* or refractive error or spectacle* or glasses or corrective lens*).tw. | 59476 |
|  | 15 | or/8-14 | 1775244 |
|  | 16 | exp economics/ or health care rationing/ or health resources/ or exp technology assessment, biomedical/ or exp models, economic/ | 680972 |
|  | 17 | (economic analys* or economic evaluation or economic model or "value for money" or monetary value).tw. | 22947 |
|  | 18 | (cost minimi* or cost comparison or cost benefit or cost effective* or cost utilit*).tw. | 185172 |
|  | 19 | or/16-18 | 808712 |
|  | 20 | 7 and 15 and 19 | 345 |
| Embase  <1974 to 13/06/2023> | 1 | exp indigenous people/ or minority group/ or Australoid/ or vulnerable population/ or exp indigenous health care/ or exp oceanic ancestry group/ or exp australian aborigine/ or caribbean person/ | 86072 |
|  | 2 | (indigenous or native or aborigin* or first nation* or first people* or islander or trib* or elder or ancest* or village* or ethnic minority).tw. | 561603 |
|  | 3 | (ATSI or Torres Strait* or Australoid or closing the gap).tw. or health services, indigenous/ | 6230 |
|  | 4 | (Polynesian or Maori or Hawaiian).tw. | 10985 |
|  | 5 | (Indian* or Amerind or Pima or Akimel O'otham or Navajo or Navaho or Dine).tw. not India/ | 96004 |
|  | 6 | (Inuit* or Inuk or Kalaallit or Inupiat or Aleut or Eskimo or Metis).tw. | 3363 |
|  | 7 | or/1-6 | 704999 |
|  | 8 | exp ophthalmology/ or exp visual system examination/ or exp intraocular drug administration/ or exp eye surgery/ or exp eye disease/ | 1186034 |
|  | 9 | (ophthal* or optometr*).tw. | 178660 |
|  | 10 | (eye or eyes).tw. | 522435 |
|  | 11 | (vision or visual* or sight or blindness).tw. | 1138803 |
|  | 12 | (teleophthalmolog* or tele ophthalmolog*).tw. | 623 |
|  | 13 | (amblyopia or aphakia or cataract or cornea* or dry eye or glaucoma or macular degeneration or macular oedema or macular edema or onchocerciasis or onchocerciases or optic or orbital or pterygium or retina or retinal or retinopathy or river blindness or trachoma or trichiasis).tw. | 705414 |
|  | 14 | (astigmatism or hyperopi* or hypermetropi* or myopi* or presbyopia* or refractive error or spectacle* or glasses or corrective lens*).tw. | 67595 |
|  | 15 | or/8-14 | 2360159 |
|  | 16 | exp health economics/ or exp health care planning/ or exp economic model/ or exp health care organization/ or exp biomedical technology assessment/ | 2712220 |
|  | 17 | (economic analys* or economic evaluation or economic model or "value for money" or monetary value).tw. | 32775 |
|  | 18 | (cost minimi* or cost comparison or cost benefit or cost effective* or cost utilit*).tw. | 256267 |
|  | 19 | or/16-18 | 2819956 |
|  | 20 | 7 and 15 and 19 | 2328 |
| Web of Science Core Collection  <1997 to 13/06/2023> |  | TS=((indigenous or native or aborigin* or "first nation" or "first nations" or "first people" or "first peoples" or islander or tribe or tribes or tribal or "ethnic minority" or atsi or "torres strait" or "torres straits" or "australoid" or "australid" or "closing the gap" or caribbean or "american indian" or "native american" or "pacific islander" or "pacific islanders" or polynesian or maori* or hawaiian or amerind or pima or "akimel" or navajo or navaho or dine or "o'otham" or inuit* or inuit or kalaallit or inupiat or aleut or eskimo or metis) and (ophthal* or optometr* or intraocular or "eye disease" or "eye diseases" or "eye injury" or "eye injuries" or eyes or vision or visual* or sight or blindness or teleophthalmolog* or "tele ophthalmology" or amblyopia or aphakia or cataract or cornea* or "dry eye" or glaucoma or "macular degeneration" or "macular oedema" or "macular edema" or onchocerciasis or onchocerciasis or optic or orbital or pterygium or retina or retinal or retinopathy or "river blindness" or trachoma or trichiasisastigmatism or hyperopi* or hypermetropi* or myopi* or presbyopia* or "refractive error" or spectacle* or glasses or "corrective lens" or "corrective lenses") and (economic* or "health resources" or "technology assessment" or "value for money" or "monetary value" or "cost minimisation" or "cost minimization" or "cost comparison" or "cost benefit" or "cost effective" or "cost effectiveness" or "cost utility" or "cost utilization" or "cost utilisation")) | 1023 |
| The Cochrane Library  <Searched Title, Abstract, Keywords to issue 6, 2023> |  | ((indigenous OR native OR aborigin* OR "first nation" OR "first nations" OR "first people" OR "first peoples" OR islander OR tribe OR tribes OR tribal OR "ethnic minority" OR atsi OR "torres strait" OR "torres straits" OR "australoid" OR "australid" OR "closing the gap" OR caribbean OR "american indian" OR "native american" OR "pacific islander" OR "pacific islanders" OR polynesian OR maori* OR hawaiian OR amerind OR pima OR "akimel" OR navajo OR navaho OR dine OR "o'otham" OR inuit* OR inuit OR kalaallit OR inupiat OR aleut OR eskimo OR metis) AND (ophthal* OR optometr* OR intraocular OR "eye disease" OR "eye diseases" OR "eye injury" OR "eye injuries" OR eyes OR vision OR visual* OR sight OR blindness OR teleophthalmolog* OR "tele ophthalmology" OR amblyopia OR aphakia OR cataract or cornea* OR "dry eye" OR glaucoma OR "macular degeneration" OR "macular oedema" OR "macular edema" OR onchocerciasis OR onchocerciasis OR optic OR orbital OR pterygium OR retina OR retinal OR retinopathy OR "river blindness" OR trachoma OR trichiasisastigmatism OR hyperopi* OR hypermetropi* OR myopi* OR presbyopia* OR "refractive error" OR spectacle* OR glasses OR "corrective lens" OR "corrective lenses") AND (economic* OR "health resources" OR "technology assessment" OR "value for money" OR "monetary value" OR "cost minimisation" OR "cost minimization" OR "cost comparison" OR "cost benefit" OR "cost effective" OR "cost effectiveness" OR "cost utility" OR "cost utilization" OR "cost utilisation")) | 157 |
| National Health Service Economic Evaluation Database  <1995 to 31/03/2015> | 1 | Exp Population Groups/ or Exp Vulnerable Populations/ or Exp Minority Groups/ | 141 |
|  | 2 | Exp Health Services, Indigenous/ | 0 |
|  | 3 | (Indigenous or native or aborigin* or first nation or first people or first nation people or islander or trib* or elder or ancest* or village* or ethnic minority).tw. | 147 |
|  | 4 | (ATSI or Torres Strait or Australoid).tw. | 0 |
|  | 5 | (Polynesian or Maori or Hawaiian).tw. | 4 |
|  | 6 | (Indian* or Amerind or Pima or Akimel O'otham or Navajo or Navaho or Dine).tw. NOT India/ | 65 |
|  | 7 | (Inuit* or Inuk or Kalaallit or Inupiat or Aleut or Eskimo or Metis).tw. | 1 |
|  | 8 | Exp Rural Health Services/ | 32 |
|  | 9 | Mobile Health Units/ | 14 |
|  | 10 | (outreach or mobile health* or mobile clinic or rural or remote).tw. | 328 |
|  | 11 | Or/1-10 | 596 |
|  | 12 | Exp Ophthalmology/ or Exp Optometry/ or Exp Diagnostic Techniques, Ophthalmological/ or Exp Ophthalmologic Surgical Procedures/ or Exp Eye Diseases/ or Exp Eye Injuries/ | 296 |
|  | 13 | (ophthal* or optometr*).tw. | 139 |
|  | 14 | (eye or eyes).tw. | 159 |
|  | 15 | (vision or visual* or sight or blindness).tw. | 649 |
|  | 16 | (teleophthalmolog* or tele ophthalmolog*).tw. | 5 |
|  | 17 | (amblyopia or aphakia or cataract or cornea* or dry eye or glaucoma or macular degeneration or macular oedema or macular edema or onchocerciasis or onchocerciases or optic or orbital or pterygium or retina or retinal or retinopathy or river blindness or trachoma or trichiasis).tw. | 330 |
|  | 18 | (astigmatism or hyperopi* or hypermetropi* or myopi* or presbyopia* or refractive error or spectacle* or glasses or corrective lens*).tw. | 22 |
|  | 19 | Or/12-18 | 892 |
|  | 20 | 11 and 19 | 44 |
| EconLit (EBSCO)  <Search ‘All Text’ from 1985 to 13/06/2023> | 1 | (Indigenous or native or aborigin* or first nation or first people or first nation people or islander or trib* or elder or ancest* or village* or ethnic minority or ATSI or Torres Strait or Australoid or Polynesian or Maori or Hawaiian or Indian* or Amerind or Pima or Akimel O'otham or Navajo or Navaho or Dine or Inuit* or Inuk or Kalaallit or Inupiat or Aleut or Eskimo or Metis or rural or remote or outreach or mobile health) | 172280 |
|  | 2 | (ophthal* or optometr* or eye* or vision or visual* or teleophthalmology or or sight or blindness or amblyopia or aphakia or cataract or cornea* or dry eye or glaucoma or macular degeneration or macular oedema or macular edema or onchocerciasis or onchocerciases or optic or orbital or pterygium or retina or retinal or retinopathy or river blindness or trachoma or trichiasis or astigmatism or hyperopi* or hypermetropi* or myopi* or presbyopia* or refractive error or spectacle* or glasses or corrective lens*) | 21639 |
|  | 3 | (economic analys* or economic evaluation or economic model or value for money or monetary value or cost analys* or cost minimi* or cost comparison or cost benefit or cost effective* or cost utilit*) | 187940 |
|  | 4 | 1 and 2 and 3 | 266 |
| GREY LITERATURE: |  |  |  |
| Australian Indigenous HealthInfoNet |  | Search in title or abstract (all results screened):   - Cost AND ophthalmology - Cost AND optometry - Cost AND eye - Cost AND eyes - Cost AND vision - Cost AND sight - Cost AND refractive error - Cost AND cataract - Cost AND retinopathy - Cost AND glaucoma - Cost AND trachoma - Economic AND ophthalmology - Economic AND optometry - Economic AND eye - Economic AND eyes - Economic AND vision - Economic AND sight - Economic AND refractive error - Economic AND cataract - Economic AND retinopathy - Economic AND glaucoma - Economic AND trachoma   Total (deduplicated) | 2  7  16  3  2  0  0  0  3  0  2  0  0  7  1  14  0  0  0  0  0  0  57 |
| Vision 2020 Australia |  | Search (all results screened):   - Cost - Economic   Total (deduplicated) | 139  104  243 |
|  |  |  |  |
| Informit | 1 | Indigenous OR native OR aborigin* OR “first nation“ OR “first people“ OR “first nation people“ OR islander OR trib* OR elder OR ancest* OR village* OR “ethnic minority“ OR ATSI OR “Torres Strait“ OR Australoid OR Polynesian OR Maori OR Hawaiian OR Indian* OR Amerind OR Pima OR “Akimel O'otham“ OR Navajo OR Navaho OR Dine OR Inuit* OR Inuk OR Kalaallit OR Inupiat OR Aleut OR Eskimo OR Metis OR rural OR remote OR outreach OR “mobile health“ | 159611 |
|  | 2 | ophthal* OR optometr* OR eye* OR vision OR visual* OR teleophthalmology OR sight OR blindness OR amblyopia OR aphakia OR cataract OR cornea* OR “dry eye“ OR glaucoma OR “macular degeneration“ OR “macular oedema“ OR “macular edema“ OR “onchocerciasis“ OR “onchocerciases“ OR optic OR orbital OR pterygium OR retina OR retinal OR retinopathy OR “river blindness“ OR trachoma OR trichiasis OR astigmatism OR hyperopi* OR hypermetropi* OR myopi* OR presbyopia* OR “refractive error“ OR spectacle* OR glasses OR “corrective lens“ OR “corrective lenses” | 55328 |
|  | 3 | “economic analysis“ OR “economic analyses” OR “economic evaluation“ OR “cost analysis“ OR “cost analyses” OR “cost-minimisation“ OR “cost-minimization“ OR “cost minimisation“ OR “cost minimization“ OR “cost comparison“ OR “cost-benefit“ OR “cost benefit“ OR “cost-effectiveness“ OR “cost effectiveness“ OR “cost-utility“ OR “cost utility“ OR “cost saving” OR “cost savings” | 3916 |
|  | 4 | 1 and 2 and 3 | 55 |
| National Bureau of Economic Research |  | Search in title, number, or keyword (all results screened):   - “Ophthalmology” - “Optometry” - “Eye disease” - “Eye care” - “Eye health” - “Eye service” - “Eye program” - “Eye screen” - “Eye screening” - “Visual health” - “Visual service” - “Visual screen” - “Visual screening” - “Vision service” - “Vision program” - “Vision screen” - “Vision screening” - “Teleophthalmology” - “Vision loss” - “Loss of vision” - “Impairment of vision” - “Low vision” - “Poor vision” - “Visual impairment” - “Visually disabled” - “Refractive error” - “Cataract” - “Retinopathy” - “Glaucoma” - “Trachoma”   Total (not deduplicated) | 65  42  16  26  4  2  0  2  2  0  3  1  1  5  4  3  3  0  10  3  4  7  11  33  1  4  145  15  77  11  500 |
| Canada’s Drug and Health Technology Agency |  | Search (all results screened)   - Aborigin* AND ophthalmology - Aborigin* AND optometry - Aborigin* AND eye* - Aborigin* AND vision - Aborigin* AND sight - Aborigin* AND refractive error - Aborigin* AND cataract - Aborigin* AND retinopathy - Aborigin* AND glaucoma - Aborigin* AND trachoma - Indigenous AND ophthalmology - Indigenous AND optometry - Indigenous AND eye* - Indigenous AND vision - Indigenous AND sight - Indigenous AND refractive error - Indigenous AND cataract - Indigenous AND retinopathy - Indigenous AND glaucoma - Indigenous AND trachoma - First nation* AND ophthalmology - First nation* AND optometry - First nation* AND eye* - First nation* AND vision - First nation* AND sight - First nation* AND refractive error - First nation* AND cataract - First nation* AND retinopathy - First nation* AND glaucoma - First nation* AND trachoma - Indian AND ophthalmology - Indian AND optometry - Indian AND eye* - Indian AND vision - Indian AND sight - Indian AND refractive error - Indian AND cataract - Indian AND retinopathy - Indian AND glaucoma - Indian AND trachoma - Inuit AND ophthalmology - Inuit AND optometry - Inuit AND eye* - Inuit AND vision - Inuit AND sight - Inuit AND refractive error - Inuit AND cataract - Inuit AND retinopathy - Inuit AND glaucoma - Inuit AND trachoma - Metis AND ophthalmology - Metis AND optometry - Metis AND eye* - Metis AND vision - Metis AND sight - Metis AND refractive error - Metis AND cataract - Metis AND retinopathy - Metis AND glaucoma - Metis AND trachoma   Total (deduplicated) | 7  1  18  14  3  1  4  7  2  0  5  0  20  23  9  1  6  11  5  0  9  0  19  20  8  1  8  11  4  0  25  0  53  50  21  12  29  28  23  0  5  0  12  12  5  1  4  5  2  0  1  0  5  6  2  0  1  0  0  0  176 |
| Institute of Health Economics |  | Search (all results screened)   - Aboriginal - Indigenous - First Nations - Indian - Inuit - Metis   Total (deduplicated) | 35  19  3  2  2  1  62 |
| International Health Technology Assessment database |  | Search in ALL fields (results in search 35 screened): |  |
|  | 1 | “Indigenous Peoples”[mhe] | 2 |
|  | 2 | "Health Services, Indigenous"[mhe] | 3 |
|  | 3 | “Minority Groups”[mhe] | 24 |
|  | 4 | (Indigenous) OR (native) OR (aborigin*) OR ("first nation") OR ("first nations") OR ("first people") OR ("first nation people") OR (islander) OR (trib*) OR (elder) OR (ancest*) OR (village*) OR ("ethnic minority") | 72 |
|  | 5 | (ATSI) OR (Torres Strait) OR (Australoid) | 1 |
|  | 6 | Polynesian | 0 |
|  | 7 | Maori | 1 |
|  | 8 | Hawaiian | 0 |
|  | 9 | (Indian*) OR (Amerind) OR (Pima) OR (Akimel O'otham) OR (Navajo) OR (Navaho) OR (Dine) NOT ("India"[mhe]) | 4 |
|  | 10 | (Inuit*) OR (Inuk) OR (Kalaallit) OR (Inupiat) OR (Aleut) OR (Eskimo) OR (Metis) | 1 |
|  | 11 | "Rural Health Services"[mhe] | 10 |
|  | 12 | "Mobile Health Units"[mhe] | 2 |
|  | 13 | (outreach) OR (mobile health*) OR (mobile clinic) OR (rural) OR (remote) | 190 |
|  | 14 | Or/1-13 | 280 |
|  | 15 | "Ophthalmology"[mhe] | 9 |
|  | 16 | "Optometry"[mhe] | 3 |
|  | 17 | "Diagnostic Techniques, Ophthalmological"[mhe] | 95 |
|  | 18 | "Ophthalmologic Surgical Procedures"[mhe] | 118 |
|  | 19 | "Injections, Intraocular"[mhe] | 22 |
|  | 20 | ("Eye Diseases"[mhe]) OR ("Eye Injuries"[mhe]) | 476 |
|  | 21 | (ophthal*) OR (optometr*) | 97 |
|  | 22 | (eye) OR (eyes) | 193 |
|  | 23 | (vision) OR (visual*) OR (sight) | 380 |
|  | 24 | Teleophthalmology | 1 |
|  | 25 | Blindness | 58 |
|  | 26 | (amblyopia) OR (aphakia) OR (cataract) OR (cornea*) OR (dry eye) OR (glaucoma) OR (macular degeneration) OR (macular oedema) OR (macular edema) OR (onchocerciasis) OR (onchocerciases) OR (optic) OR (orbital) OR (pterygium) OR (retina) OR (retinal) OR (retinopathy) OR (river blindness) OR (trachoma) OR (trichiasis) | 473 |
|  | 27 | (astigmatism) OR (hyperopi*) OR (hypermetropi*) OR (myopi*) OR (presbyopi*) OR (refractive error) OR (spectacle*) OR (glasses) OR (corrective lens*) | 55 |
|  | 28 | Or/15-27 | 850 |
|  | 29 | "Costs and Cost Analysis"[mhe] | 1542 |
|  | 30 | (economic analys*) OR (economic evaluation) | 1356 |
|  | 31 | (cost-minimisation) OR (cost-minimization) OR (cost minimisation) OR (cost minimization) OR (cost comparison) OR (cost-benefit) OR (cost benefit) OR (cost-effectiveness) OR (cost effectiveness) OR (cost-utility) OR (cost utility) OR (cost saving*) | 3220 |
|  | 32 | Or/29-31 | 4077 |
|  | 33 | 14 and 28 | 10 |
|  | 34 | 14 and 32 | 87 |
|  | 35 | 33 or 34 | 93 |
| The International Agency for the Prevention of Blindness |  | Search (all results screened)   - Cost - Economic - Indigenous - Aboriginal   Total (not deduplicated) | 346  363  84  51  844 |
| Google Scholar |  | (ophthalmology OR optometry OR eye OR teleophthalmology OR cataract OR macular OR retinopathy OR refractive OR trachoma) AND (economic OR cost) AND (indigenous OR native OR aboriginal OR first nation OR Maori OR Hawaiian OR American Indian)   (First 200 results screened) | 200 |

# **Appendix C:** Summary of cost-only studies on Indigenous eye care services identified during our systematic search.

| Author (year) | Intervention | Main Finding |
| --- | --- | --- |
| Lawerson (1994)^3^ | Discover Diabetes Project: annual mobile DR screening service in rural New Zealand using fundus photographs graded by an offsite ophthalmologist. Strategies to improve access for Māori people included offering screening on marae (communal place) or in houses which had been blessed. | Unadjusted cost per screen: $25-35 depending on service load. |
| Arnold (2005)^4^ | Part of Alaska Blind Child Discovery Project: pre-school photoscreening service in four urban and nine rural areas in Alaska (USA) to identify children at risk of amblyopia. | Unadjusted cost per screen: $9.15 (urban) and $15.92 (rural). Unadjusted cost per case of amblyopia detected: $182.83 (urban) and $256.39 (rural). |
| Lang (2007)^5^ | Part of Alaska Blind Child Discovery Project: vision disorder screening service for children within rural native villages in Alaska (USA) using visual acuity, polaroid photoscreener, digital photoscreener, or handheld autorefractor. | Unadjusted cost per screen: $0.56 (visual acuity), $1.12 (digital photoscreener), $56 (autorefractor), $58 (polaroid photoscreener). |
| Ralph-Campbell (2007)^6,7^ | Mobile Diabetes Screening Initiative: mobile service travelling to 25 Metis, First Nation, and other rural communities in Alberta (Canada), screening for diabetes and cardiovascular disease and complications, and providing counselling on risk minimisation. Included measurements of BMI, waist circumference, blood pressure, blood glucose and lipids, haemoglobin A1c, proteinuria and, if diagnosed with diabetes, assessment for diabetic foot disease and DR through fundus photographs (with unclear grading methods). | Unadjusted cost per patient: $887.76. |
| Chen (2015)^8^ | Mobile Vision Van Unit project: general optician and ophthalmology service for 28 Indigenous villages and rural townships in eastern Taiwan. Included an autorefractometer, retinoscope, optical dispensing workshop, tonometer, slit lamp, and ophthalmoscope. Conducted vision screening, primary eye care, and outreach specialist care. | Unadjusted cost per year: US$20,621. Unadjusted cost per patient screened: US$4.09. |

DR = diabetic retinopathy; USA = United States of America; BMI = body mass index

# **References**

1. The World Bank. World Bank Country and Lending Groups [online]. 2023. https://datahelpdesk.worldbank.org/knowledgebase/articles/906519-world-bank-country-and-lending-groups (accessed 21 April 2023).

2. United Nations. Standard country or area codes for statistical use (M49). New York: UN Statistics Division 2019.

3. Lawrenson RA, Dunn PJ, Worsley D, et al. Discover diabetes: a community based screening programme for diabetic eye disease. N Z Med J 1994; 107: 172-4.

4. Arnold RW, Armitage MD, Gionet EG, et al. The cost and yield of photoscreening: impact of photoscreening on overall pediatric ophthalmic costs. J Pediatr Ophthalmol Strabismus 2005; 42: 103-11.

5. Lang D, Leman R, Arnold AW, et al. Validated portable pediatric vision screening in the Alaska Bush. A VIPS-like study in the Koyukon. Alaska Med 2007; 49: 2-15.

6. Ralph-Campbell K, Oster RT, Connor T, et al. Emerging longitudinal trends in health indicators for rural residents participating in a diabetes and cardiovascular screening program in northern Alberta, Canada. Int J Family Med 2011; 2011: 596475.

7. Howard Research and Management Consulting Inc. Mobile Diabetes Screening Initiative evaluation 2004–2006 - final report. 2007.

8. Chen N, Hsieh HP, Tsai RK, et al. Eye care services for the populations of remote districts in eastern Taiwan: a practical framework using a Mobile Vision Van Unit. Rural Remote Health 2015; 15: 3442. doi: 10.22605/RRH3442
