# Supplementary material for: Economic evaluations of eye care services for Indigenous populations in high-income countries: a scoping review
Source: Int J Equity Health. 2024 Nov 9;23:232. doi: 10.1186/s12939-024-02307-z (PMC11549826; doi:10.1186/s12939-024-02307-z)
Supplement: Supplementary file 2 — Supplementary Material 2. [file 12939_2024_2307_MOESM2_ESM.docx]

**Supplementary Material**

**Table of Contents**

[**Supplementary Table 1.** Quality scores of each study using the QHES Checklist 2](#_Toc171698837)

[**Supplementary Table 2.** Types of costs and outcomes included in economic evaluations of Indigenous eye care services. 3](#_Toc171698838)

[**References** 7](#_Toc171698839)

| Author (Year) | 1 | 2 | 3 | 4 | 5 | 6 | 7 | 8 | 9 | 10 | 11 | 12 | 13 | 14 | 15 | 16 | Score (%) |
| --- | --- | --- | --- | --- | --- | --- | --- | --- | --- | --- | --- | --- | --- | --- | --- | --- | --- |
| Griffith (1993) | N | N | Y | NA | N | NA | Y | N | N | Y | NA | Y | N | N | Y | N | 49 |
| Jaworski (1996) | Y | Y | Y | NA | N | Y | Y | Y | Y | N | N | Y | Y | N | Y | N | 69 |
| Martin (1998) | Y | N | N | NA | N | NA | N | N | N | Y | NA | N | N | N | N | N | 27 |
| Miller (2003) | Y | N | N | NA | N | NA | Y | N | N | Y | NA | Y | N | N | Y | Y | 51 |
| Maberley (2003) | Y | Y | Y | NA | Y | Y | Y | Y | N | Y | N | Y | Y | N | Y | N | 76 |
| Jin (2004) | Y | N | Y | NA | N | NA | Y | N | N | Y | NA | Y | N | N | N | N | 48 |
| Whited (2005) | Y | Y | N | Y | Y | Y | Y | N | Y | Y | Y | Y | Y | Y | Y | Y | 85 |
| Ho (2006) | Y | N | Y | NA | Y | NA | Y | Y | Y | Y | NA | Y | N | N | N | N | 72 |
| Kumar (2006) | Y | Y | Y | NA | Y | NA | Y | N | Y | Y | NA | N | N | N | N | Y | 64 |
| Turner (2011)a | Y | N | Y | NA | N | NA | Y | Y | N | Y | NA | Y | N | N | Y | Y | 66 |
| Turner (2011)b | Y | N | Y | NA | N | NA | Y | Y | N | Y | NA | Y | N | N | N | Y | 58 |
| FNQLHSSC (2013) | Y | N | Y | NA | N | NA | Y | Y | N | Y | NA | Y | N | Y | N | Y | 64 |
| Ellery (2014) | Y | Y | Y | Y | Y | Y | Y | Y | Y | Y | N | Y | N | Y | Y | Y | 86 |
| PWC (2015) | Y | Y | Y | NA | Y | Y | Y | N | N | Y | Y | Y | Y | Y | Y | Y | 85 |
| Kanagasingam (2015) | N | N | N | NA | N | NA | N | N | N | N | NA | N | N | N | N | Y | 17 |
| Kim (2015) | Y | N | Y | NA | N | NA | Y | N | N | Y | NA | Y | N | N | N | Y | 51 |
| Razavi (2016) | Y | Y | Y | NA | N | NA | Y | Y | Y | Y | NA | Y | Y | N | Y | Y | 85 |
| Ballreich (2016) | Y | Y | N | NA | Y | Y | N | N | Y | N | N | Y | N | Y | Y | N | 57 |
| Kanjee (2017) | Y | Y | Y | NA | N | NA | Y | N | N | Y | NA | Y | N | N | Y | Y | 63 |
| Stanimirovic (2019) | Y | Y | N | NA | Y | Y | Y | Y | N | Y | N | Y | N | N | N | Y | 56 |

# **Supplementary Table 1. Quality scores of each study using the QHES Checklist**

N= no; Y = yes; NA = not applicable

# **Supplementary Table 2. Types of costs and outcomes included in economic evaluations of Indigenous eye care services.**

| Author (year) | Design | Costs | | | | | Outcome | Other Concerns |
| --- | --- | --- | --- | --- | --- | --- | --- | --- |
|  |  | Capital | Staff Wages | Staff  Travel | Treatment | Others |  |  |
| DR Screening  (Health Clinics) |  |  |  |  |  |  |  |  |
| Griffith (1993) | CMA | No | Yes | NA | NA | “Material costs” | NA |  |
| Maberley (2003) | CEA, CUA | Yes | Partial^a^ | NA | No |  | Cases of STDR detected  Years of blindness avoided QALYs gained | Overestimated annual incidence of blindness from macular oedema of 10% (untreated) and 4% (PRP), thus overestimating blindness avoided by intervention and comparator. |
| Whited (2005) | CEA | Yes | Yes | NA | Yes | Customer support  Federal cost of vision loss | Cases of STDR detected Cases of blindness avoided | Did not include outcomes extending beyond the one-year horizon. |
| FNQLHSSC (2013) | CMA | No | Partial^b^ | NA | Yes^c^ | Patient/carer travel and productivity losses (for ophthalmology consult or treatment) | NA | Sub-section of report estimated additional capital and operational costs at $47,324 per year. Including these makes intervention more expensive ($1,270 per patient) than screening by out-of-town optometry ($1,120) or  ophthalmology ($948). |
| Ellery (2014) | CEA, CUA | Yes^d^ | Yes^d^ | NA | Yes | Maintenance  Consumables  Training  Healthcare costs of different DR severities | Cases of blindness avoided QALYs gained | Omitted travel costs for rural patients with no local optometrist or ophthalmologist, thus underestimated cost of this comparator.  Blindness avoided based on treatment of proliferative DR (not macular oedema), but number treated included both, thus overestimating blindness avoided by intervention and comparators. |
| Kanagasingam (2015) | CMA | Unclear^e^ | Yes | NA | NA | Patient travel (comparator only) Data connection | NA | Underestimated costs for screening by out-of-town ophthalmology (only included patient travel costs) or outreach ophthalmology (omits nursing and clerical costs) comparators, thus underestimating savings from intervention. |
| Ballreich (2016) | CEA | Yes | Yes | NA | Yes^c^ | Patient travel to GP/optometrist | Cases of DR detected | Cost of optometry based on average government reimbursement in very remote areas ($275) rather than average across all rural areas ($183), thus overestimating cost of comparator.  Omitted savings from comparator from extra cases of DR treated and vision loss avoided. |
| DR Screening (Mobile) |  |  |  |  |  |  |  |  |
| Martin (1998) | CMA | Yes | Partial^f^ | Yes | NA | Patient travel (comparator only)  Maintenance  Insurance | NA |  |
| Jin (2004) | CMA | Yes | Unclear^g^ | Unclear^g^ | NA | Patient travel (comparator only) | NA |  |
| Ho (2006) | CMA | Yes | Yes | Yes | NA | Maintenance  Consumables  Data connection |  |  |
| Kim (2015) | CMA | Unclear^h^ | Yes | Yes | NA | Patient/carer travel (comparator only) Consumables |  |  |
| Kanjee (2017) | CMA | Unclear^i^ | Yes | Yes | NA | Patient travel (comparator only)  Training |  |  |
| Stanimirovic (2019) | CEA | Yes | Yes | Yes | NA | Maintenance  Consumables | Cases of DR detected | Likely overestimated cost-effectiveness of intervention: (a) costs based on screening 28,500, but DR cases calculated assuming only 22,800 and 15,675 attend screening in intervention and comparator, respectively; (b) comparator is an ophthalmology consult, which has a higher sensitivity and specificity than intervention^1^ (despite analysis allocating it lower values). |
| General Telehealth |  |  |  |  |  |  |  |  |
| Kumar (2006) | CMA | Yes | Yes | NA | NA | Patient travel (comparator only)  Training  Data connection  Marketing | NA |  |
| Razavi (2016) | CMA | Yes | Yes | NA | NA | Patient travel and productivity losses including tax revenue forgone (comparator) | NA |  |
| General Outreach |  |  |  |  |  |  |  |  |
| Turner (2011) | CMA | Unclear^j^ | Unclear^j^ | Unclear^j^ | Unclear^j^ |  | NA |  |
| Turner (2011) | CMA | Unclear^j^ | Unclear^j^ | Unclear^j^ | Unclear^j^ |  | NA |  |
| Others |  |  |  |  |  |  |  |  |
| Jaworski (1996) | CBA | Yes | Yes | NA | Yes | Maintenance Consumables  Rent  Utilities | BCR | Benefits based on amount insurance parties are billed, not actual amount received. |
| Miller (2003) | CMA | Yes | No^k^ | NA | NA | Diagnostic eye exam for positive screens | NA |  |
| PwC (2015) | CBA | Partial^l^ | Yes | Yes | Yes | Patient travel if rural | BCR |  |

DR = diabetic retinopathy; CMA = cost-minimisation analysis; NA = not applicable; CEA = cost-effectiveness analysis; CUA = cost-utility analysis; STDR = sight-threatening diabetic retinopathy; QALY = quality-adjusted life years; PRP = panretinal photocoagulation; GP = general practitioner; CBA = cost-benefit analysis; BCR = benefit-cost ratio; PwC = PricewaterhouseCoopers
^a^ Included cost of technician acquiring photographs, but unclear who read photos and if reading costs were included.
^b^ Included offsite photo reading cost, but omitted cost of technicians and nurse conducting screening.
^c^ Included treatment costs for both service and comparator without analysing outcomes of treatment.
^d^ Each screen attracted a government cost (‘Medicare Benefits Schedule fee’) of $42.25, 40% of which covered capital and training. This fee was
 proposed as it would cover capital costs in 5 years, assuming 10 screens per week.
^e^ Included cost of ‘camera depreciation’ but unclear if it was annuitized or if other capital costs were included.
^f^ Included cost of technician acquiring photographs but omitted cost of local nurses assisting the service.
^g^ Included total operating cost of service but the specific components unstated.
^h^ Unclear what components were included in capital costs and whether these were annuitized.
^i^ Includes cost of ‘equipment’ but unclear if this was annuitized or if other capital costs were included.
^j^ Includes ‘total costs relating to the service from State, Commonwealth and other sources’, but does not specify the items included. Unclear how capital
 costs were integrated into reported ‘cost per attendance’.
^k^ Only compares capital cost of screening by autorefraction, autokeratometry, photoscreening, or visual acuity and the cost of diagnostic exams for positive
 screens – assumes all other costs are equal between different screening programs.
^l^ Each consult attracts a government cost, a portion of which represents capital costs. However, omitted total upfront capital costs.

# **References**

1. Mehraban Far P, Tai F, Ogunbameru A, et al. Diagnostic accuracy of teleretinal screening for detection of diabetic retinopathy and age-related macular degeneration: a systematic review and meta-analysis. BMJ Open Ophthalmol 2022; 7: e000915. doi: 10.1136/bmjophth-2021-000915
